# Autophagy in Atherosclerotic Plaque Cells: Targeting NLRP3 Inflammasome for Self-Rescue

**DOI:** 10.3390/biom13010015

**Published:** 2022-12-21

**Authors:** Xuelian Li, Xianjie Zhu, Yumiao Wei

**Affiliations:** 1 Department of Cardiology, Union Hospital, Tongji Medical College, Huazhong University of Science and Technology, Wuhan 430022, China; 2 Department of Orthopaedic Surgery, Qingdao Municipal Hospital, Qingdao 266011, China

**Keywords:** autophagy, mitophagy, NLRP3 inflammasome, atherosclerosis

## Abstract

Atherosclerosis (AS) is a lipid-driven disorder of the artery intima characterized by the equilibrium between inflammatory and regressive processes. A protein complex called NLRP3 inflammasome is involved in the release of mature interleukin-1β (IL-1β), which is connected to the initiation and progression of atherosclerosis. Autophagy, which includes macroautophagy, chaperone-mediated autophagy (CMA), and microautophagy, is generally recognized as the process by which cells transfer their constituents to lysosomes for digestion. Recent studies have suggested a connection between vascular inflammation and autophagy. This review summarizes the most recent studies and the underlying mechanisms associated with different autophagic pathways and NLRP3 inflammasomes in vascular inflammation, aiming to provide additional evidence for atherosclerosis research.

## 1. Introduction

Atherosclerosis (AS) is a chronic disease marked by atherosclerotic plaque formation in arterial walls that wholly or partially occlude the vessel. Usually, an inner layer of blood vessels is lined with vascular endothelial cells (ECs), which regulate the tone of the blood vessels and protect them from inflammation and thrombosis [1]. The onset of AS begins with EC dysfunction due to disturbed blood flow and accumulated lipids. Afterward, damaged endothelial cells release adhesion molecules and chemokines to recruit circulating monocytes. Infiltrating monocyte-derived macrophages and resident macrophages consumed oxidized low-density lipoprotein (ox-LDL), forming foam cells [2]. The inflammatory cytokines released by macrophages results in the de-differentiation of vascular smooth muscle cells (VSMCs) into migratory and macrophage-like foam cells [3]. Atherosclerosis-related plaque instability is caused by the death of macrophage-derived or VSMC-derived foam cells within advanced lesions [4]. A ruptured atherosclerotic plaque, and any subsequent thrombosis event, can result in complex clinical complications, such as myocardial infarction [5,6] and stroke [7]. Recent studies have revealed that autophagy is crucial for early and advanced atherosclerosis formation. Using mouse and human specimens, the autophagy markers LC3-II and p62 were found in plaque cells, indicating dysfunctional or decreased autophagy [8,9]. Mitophagy controls mitochondrial quality and quantity by eliminating damaged mitochondria, and its disruption plays an instrumental role in atherosclerosis. Moreover, studies focusing on chaperone-mediated autophagy (CMA) in atherosclerosis have recently been published [10,11].

Activated innate immune systems often cause maladaptive inflammation that promotes the remodeling and dysfunction of the cardiovascular system [12]. An important component of the immune system is called a TLR (toll-like receptor). Atherosclerosis can be accelerated by TLR ligands, and subverted TLR signaling can slow down the progression of the disease. Inflammasomes cause inflammation across a wide range of pathological conditions by detecting changes within the cell. The most well-known inflammasome, NLRP3, is essential for atherogenesis, and its silencing stabilizes atherosclerotic plaque [13]. The Canakinumab Anti-Inflammatory Thrombosis Outcome Study (CANTOS) test [14] revealed that the anti-inflammatory therapy targeting the interleukin-1β (IL-1β) pathway remarkably cut the recurrence rate of cardiovascular incidents, regardless of lipid level. Since the NLRP3 inflammasome is involved in the inflammatory autophagic cross-talk, intensive research into its roles in AS and medicinal interventions are essential for the treatment and prevention of AS. In this review, the effects of autophagy and the NLRP3 inflammasome on AS and their underlying mechanisms will be systematic reviewed to provide a theoretical framework for future AS research.

In this review, we focus on the detailed mechanisms governing cargo recognition in defined mammalian autophagy pathways. Further, we provided an overview of the most recent findings regarding the relationship between defective autophagy and NLRP3 inflammasomes in the etiopathogenesis of atherosclerosis.

## 2. The Overview of Autophagy

During autophagy, proteins and damaged organelles are removed due to folding errors, which is a type of intracellular degradation process. There are three types of autophagy based on our current knowledge, macroautophagy, chaperone-mediated autophagy, and microautophagy, which differ primarily in terms of cargo delivery to the lysosome. In macroautophagy, cytoplasmic contents are sequestered into a double-walled membrane and then fused to the lysosome. By using signal peptides, CMA selectively localizes proteins to the lysosomes and coordinates them with chaperones located both inside and outside the target membrane. A microautophagic process is defined by the lysosomes directly engulfing cytoplasmic cargo. For maintaining cellular homeostasis, select autophagy, including mitophagy, lipophagy, xenophagy, and ERphagy, are engaged for clearing mitochondria, lipid droplets, invading pathogens, and endoplasmic reticulum. Our discussion here focuses on macroautophagy, CMA, and the major selective autophagy (mitophagy); microautophagy is covered elsewhere in more detail [15].

### 2.1. Macroautophagy

**Figure 1:**
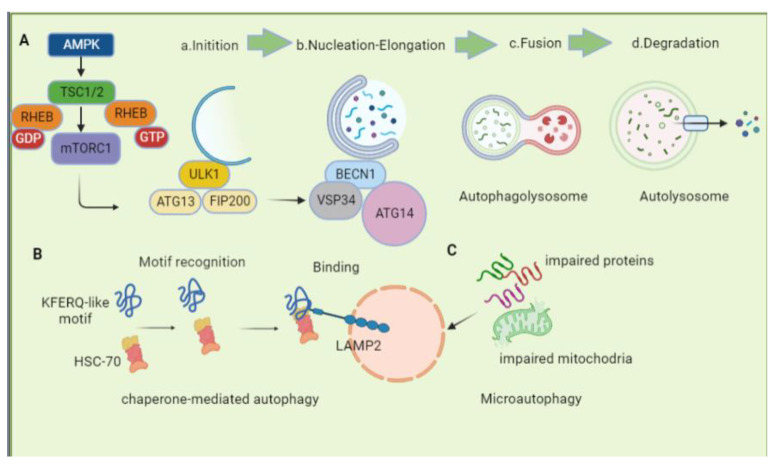
Schematic representation of different autophagy pathways. (**A**). macroautophagy: (a) Initiation, the initiation of autophagy is regulated by ULK1-FIP200-ATG13 complex. (b) Nucleation-Elongation, during the phagophore formation, Vps34-BECN1-ATG14 is recruited, and the cytoplasm and organelles are wrapped and devoured; (c) Fusion, fusion and docking of the autophagosome with the lysosome. (d) Degradation, interior autolysosome cargo degradation. (**B**). Mechanism and regulation of CMA. CMA begins with the recognition of a substrate protein bearing a KFERQ-like motif by HSC70 in the cytosol. In CMA, binding of the HSC70/substrate complex to LAMP2A at the lysosomal membrane causes LAMP2A to multimerize and form a translocation complex, which promotes the internalization of the substrate protein into the lumen for destruction. CMA, chaperone-mediated autophagy; HSC70, Heat shock cognate protein 70 KDa; LAMP-2A, lysosome-associated membrane protein type 2A. (**C**). Microautophagy: Microautophagy involves the direct uptake of autophagic cargoes by lysosomes, which are then degraded in endolysosomes.

Molecular components of macroautophagy were first identified in yeast with the discovery of ATG5 [16], ATG3 [17], ATG13 [18], and ATG1 [19]. Over 40 ATG proteins have been identified to control the various stages of autophagy in yeast (most of which have homologs in mammalian cells and plants). A multi-step autophagic process includes the induction of autophagy, the formation of autophagosomes, the docking and fusion of autophagosomes with lysosome membranes, and the degradation and recirculation of autophagosome internal contents (Figure 1).

#### 2.1.1. Induction

An autophagy process begins with the activation of the ULK1 complex. The mTOR complex 1 (mTORC1) and AMPK regulate ULK1 kinase activity [20]. The mTOR is a serine/threonine protein kinase that belongs to the PI3K-related protein kinase (PIKK) family and is responsible for regulating autophagy [21]. There are two distinct forms of mTOR in mammals: mTORC1 and mTORC2. The mTOR complex enhances ATP production under energy-deficient conditions by linking autophagic activity to energy conditions. Tuberous sclerosis complex (TSC) regulates the activity of mTORC1 by acting as the GTPase-activating protein (GAP) for the small GTPase Rheb. TSC is a heterogeneous complex with members TSC1, TSC2, and Tre2-Bub2-Cdc16 (TBC) 1 domain family, member 7 (TBC1D7) [21,22]. As nutrient levels decrease, mTORC1 has a tendency to dissociate from ULK1, which is followed by ULK1 being dephosphorylated, which initiates autophagy. In regulating cellular energy balance, AMPK plays an important role as an energy-sensing kinase. AMPK can be triggered by surplus AMP and ADP or by deficiency of ATP caused by glucose starvation. The induction of autophagy by AMPK is dependent upon ULK1-specific ser555 residues [23]. As low-energy states activate AMPK, TSC2 is phosphorylated, repressing mTOR, which leads to a cross-regulation between AMPK and mTOR activity [24,25]. Mammals have a ULK1 complex composed of ULK1, as well as the non-catalytic subunits FIP200, ATG101, and ATG13 [26,27,28]. To activate the ULK1/ATG1 kinase complex, Thr180 in human ULK1 or Thr226 in yeast ATG1 must be autophosphorylated [29]. Induced autophagy conditions, as well as the co-assembly of other subunits, promote autophosphorylation. In turn, this co-assembly promotes mutual autophosphorylation of ATGl molecules and increases their local concentration. FIP200 functions as scaffolding for downstream ATG protein assembly at the pre-autophagosomal structure (PAS), while ATG13 contributes to ULK1’s PAS localization and interaction with FIP200 [30,31]. The initiation of autophagy occurs once ATG13 and ULK1 bind to the PAS, engaging and localizing ATG proteins.

#### 2.1.2. Nucleation, Elongation, and Maturation

Both yeast and mammals rely on the PtdIns3K complex to initiate phagophore nucleation. It consists of PtdIns3KVPS34, ATG14/mATG14, VPS15/p150, and VPS30/BECN1. BECN1 regulates class III PI3K complex I and, by binding to certain proteins, it can trigger or inhibit autophagy [32]. As a result of binding to BECN1, an activating molecule in BECN1-regulated autophagy protein 1 (AMBRA1) promotes the interaction of BECN1 with VPS34, and, consequently, autophagy is activated. Moreover, through interactions with RUBICON, ATG14L [33], UVRAG [34], and VMP1 [35], BECN1 regulates membrane formation. PtdIns III K produces phosphatidylinositol-3-phosphate (PtdIns3P), a phosphatidylinositol that serves as a hallmark for the recruitment of components involved in autophagosome formation. WD repeat domain phosphoinositide-interacting (WIPI) protein is involved in membrane elongation at the PI3P synthesis site [36,37]. The expansion of autophagosomes is mediated by two systems: (1) ATG12-ATG5 system, and (2) ATG8/LC3 system [38]. Other subfamilies of ATG8 paralogs in mammals, such as GABARAP, GABARAPL1, and GABARAPL2/GATE-16, may regulate autophagosome maturation during a later step [39].

#### 2.1.3. Fusion and Degradation

Upon arriving at its destination, the autophagosome must fuse with the endocytic system. The SNARE (soluble N-ethylmaleimide-sensitive factor attachment protein receptor) complex mediates autophagosome-lysosome fusion. The motifs Qa, Qb, Qc, and R that each SNARE provides form a four alpha-helix bundle during SNARE-mediated membrane fusion [40]. As tethering factors are recruited to target membranes or vesicles during fusion, SNARE proteins and small RAB GTPases are essential for capturing vesicles and forming trans-SNARE complexes [41,42]. SNAREs disassemble after fusion and return back to their compartments to maintain intracellular membrane identity [43]. A lysosome fused with an autophagosome forms an autophagolyosome, which contains many enzymes in lysosomes called lysosomal hydrolases. Those enzymes can break down autophagosome inner membranes and macromolecules from the cytoplasm into amino acids or peptides that can be reused by the cells [44].

### 2.2. Chaperone-Mediated Autophagy

In the delivery of cargo via CMA, no intermediate vesicles are formed, and no membrane fusion or deformation occur [45,46]. Selective CMA involves three distinct steps: (i) recognizing and translocating substrates to lysosomes; (ii) interacting with receptors and unfolding; and (iii) lysosome-mediated translocation and degradation. To recognize CMA substrates, the constitutive chaperone, heat shock cognate protein 70 KDa (HSC70), combines with a pentapeptide motif called KFERQ in substrate proteins [47]. One or two positively charged residues (K and R) are present in this motif, as well as one or two hydrophobic residues (I, L, V, F), one negative charge (D, E), and one glutamine (Q) on either side. The KFERQ motif is found in about 30% of cytosolic proteins, but by phosphorylation and acetylation, KFERQ-like motifs can be created, increasing the number of proteins degraded by CMA [47,48,49]. Upon binding CMA substrates, cytosolic chaperones deliver them to lysosomes. There, a substrate protein/chaperone complex binds to the single-span membrane protein, lysosome-associated membrane protein type 2A (LAMP-2A). There are three splice variants of the *Lamp2* gene, all of which share the same luminal region but have different transmembrane and cytosolic tails [50]. LAMP-2A binding limits CMA and, therefore, cells can rapidly upregulate or downregulate CMA by changing the levels of this membrane protein [51]. Upon translocation into the lysosome lumen, the substrate is broken down into amino acids by the relevant hydrolase, providing the cell with the amino acids required for protein synthesis [45].

### 2.3. Microautophagy

During microautophagy, cytoplasmic entities (in yeasts and plants) are taken up directly by the vacuole through membrane invagination or by endo-lysosomal compartments in mammals. In addition to proteins, microautophagy cargoes include mitochondria, peroxisomes, and portions of nuclear fragments [52,53]. Endosomal microautophagy (eMI) occurs in mammalian cells where cytosolic proteins are degraded in late endosomes/multivesicular bodies and are further subdivided according to cargo selectivity [54,55]. These proteins contain a KFERQ-like motif that is recognized by HSC70 to facilitate the translocation of captured proteins into the lumen of a late endosome, or multivesicular body, via the endosomal sorting complex required for transport (ESCRT) III [56]. In comparison to macroautophagy and CMA, microautophagy is much less understood, so this review will not provide a detailed explanation.

### 2.4. Selective Autophagy: Mitophagy

**Figure 2:**
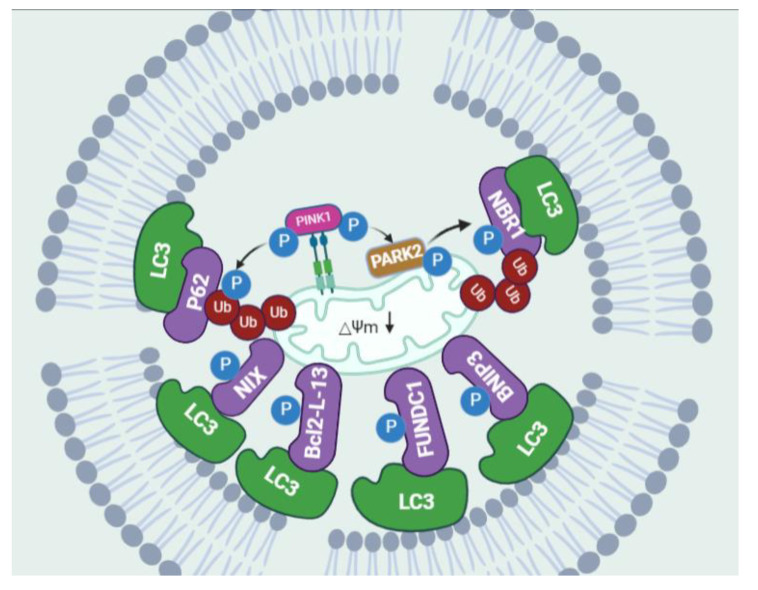
Molecular mechanism of mitophagy. A decreased mitochondrial membrane potential increases membrane depolarization and accumulates PINK1 kinase at the OMM. PINK1 recruits PARK2 and targets it at the damaged mitochondria. The activation of PINK1/PARK2 in the OMM allows the interaction with LC3 for autophagic degradation through specific adaptor proteins, such as SQSTM1/p62, which binds simultaneously with ubiquitin and LC3 to drive the process. Mitophagy is also induced by mitochondrial receptors, such as BNIP3, NIX, FUNDC1, Bcl2-L-13, which directly bind LC3 through the conserved LC3-interacting region (LIR).

The term “mitophagy”, first coined by Priault et al. [57], has led to major breakthroughs in recent years, with the discovery of key proteins that selectively mediate mitochondrial degradation in yeast and mammalian cells. Mitophagy is typically classified into two separate categories according to the necessity for the Ub E3 ligase PARK2 and the kinase PINK1; these two groups are commonly known as PINK1/PARK2-dependent and independent mitophagy [58]. PINK1/PARK2-dependent mitophagy is the primary process in the cell for the removal of depolarized mitochondria. PINK1 is a constitutively imported mitochondrial protein kinase that is cleaved by the PARL [59,60]. However, damaged mitochondria with a diminished membrane potential are incapable of importing PINK1 to the inner mitochondrial membrane (IMM), leading to the stability of PINK1 on the outer mitochondrial membrane (OMM) and the subsequent phosphorylation of ubiquitin and recruitment of PARK2 [61,62,63]. A polyubiquitin chain is formed by active PARK2, which, in turn, recruits autophagic cargo receptor proteins, such as SQSTM1/p62, NBR1 (autophagic cargo receptor NBR1), and OPTN (optineurin) [64,65,66]. These receptors simultaneously bind to light chain 3B (LC3B) on autophagosome membranes, leading to the engulfment of targeted mitochondria within autophagosomes [64]. Aside from Ub-driven mitophagy, a number of other mitophagy mechanisms have now been reported that target mitochondria to the autophagosome in a non-ubiquitin-dependent manner. These mitophagy receptors, such as BCL2 interacting protein 3 (BNIP3) [67], NIP3-like protein X (NIX) [68], Bcl2-like protein 13 (Bcl2-L-13) [69], and FUN14 domain containing protein 1 (FUNDC1) [70], respond to stimuli to fine-tune mitochondrial populations. Through canonical or atypical LC3-interacting region (LIR) motifs, these mitophagy receptors are capable of directly interacting with LC3 and/or a GABA-receptor-associated protein (GABARAP). In response to hypoxia, HIF hypoxia-inducible factor-1 (HIF-1) and/or forkhead homeobox type O (FOXO) induce the transcription of BNIP3 and NIX, inducing autophagy to eliminate damaged mitochondria [71]. The integrity and function of mitochondria-associated ER membranes (MAMs), as well as the functional and physical contact between mitochondria and ER, are controlled by FUNDC1 to facilitate mitophagy [72,73] (Figure 2).

The autophagy process has traditionally been known as a bulk recycling of intracellular components but has now emerged as a coordinated adaptative pathway that maintains cellular viability under a variety of stress-related conditions, including starvation, hypoxia, metabolic abnormalities, and protein aggregation. Many diseases are correlated with autophagy, including heart disease, cancer, and neurodegenerative diseases, due to changes in the sensitivity of cells to death. Even though great progress has been made in understanding the mechanism and regulation of autophagy, many questions remain.

## 3. Role of Autophagy in Atherosclerosis

**Figure 3:**
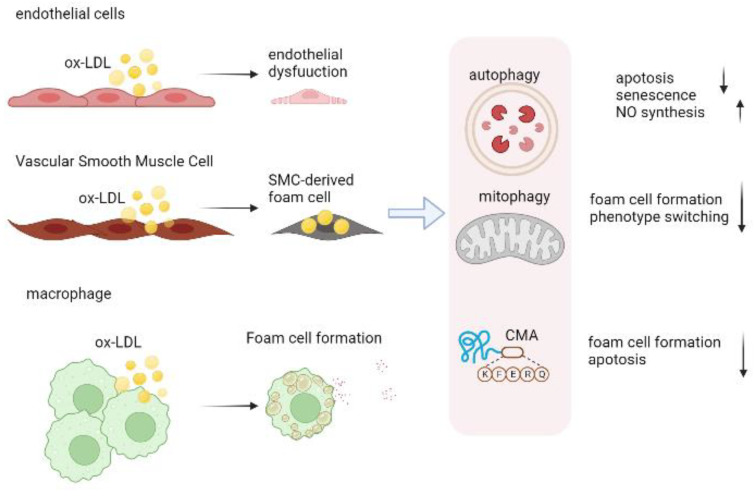
Impact of cell-specific autophagy on the atherosclerotic plaque formation. In ECs, autophagy enhances NO bioavailability and prevents endothelial apoptosis and senescence in atherosclerotic plaque. Autophagy regulates the proliferation of vascular smooth-muscle cells, contributes to their phenotypic switch, and inhibits the VSMCs-derived foam cell formation. Additionally, autophagy activation can inhibit foam cell formation and lipid-laden macrophage apoptosis. ECs, endothelial cells; NO, nitric oxide; VSMCs, vascular smooth-muscle cells.

Multiple cells are engaged in the pathophysiology of atherosclerosis, including endothelial cells, immune cells, and VSMCs (Figure 3). As atherosclerosis progresses, autophagy profoundly influences the behavior of these cells. Multifactorial mechanisms are involved in atherosclerosis, and various pro-atherogenic factors (e.g., cytokine release, oxidized lipids, hypoxia) are all known to stimulate autophagy. The autophagy process may be a process for recycling damaged cellular components in atherosclerosis plaques to promote cell survival, and for this reason, autophagy in atherosclerosis is traditionally regarded as beneficial. By degrading polarized mitochondria before their release of cytochrome c, autophagy prevents plaque cells from suffering from oxidative stress, an occurrence that leads to advanced atherosclerosis [74]. By inhibiting apoptosis and facilitating cellular recovery, autophagy also inhibits the development of apoptosis. The endothelium responds to external stimuli by regulating autophagy, which then counters inflammatory responses, oxidative stress, and thrombotic factors. Additionally, pretreatment with rapamycin significantly reduced VSMC cell death, thereby enhancing plaque stability, suggesting autophagy is crucial for VSMC survival [75]. A powerful approach to inhibit or reverse atherosclerosis may be to improve macrophage autophagy by promoting cholesterol efflux and suppressing inflammation, since macrophage autophagy plays a key role in proatherogenic factors.

### 3.1. Autophagy in Endothelial Cells

Endothelial apoptosis and damage cause the weakening of the endothelial barrier and accumulate adhesion proteins, which are early stepping stones in AS. The protective role of autophagy appears to occur in the first stages of atherosclerosis in order to shield endothelial cells from oxidized lipids, metabolic stress, and inflammation. As a result, cells survive and atherosclerosis progression is inhibited [76,77]. The deletion of the endothelial-specific *Atg5* and *Atg7* genes raises atherosclerotic burdens in Apoe^−/−^ mice [78,79]. Moreover, TUNEL-positive nuclei were noted in endothelial *Atg5*-deficient mice, indicating increased endothelial apoptosis [80]. Endothelial nitric oxide (NO) blocks platelet adhesion molecules, growth factors, and cytokines produced by ECs in the vascular wall, which are inflammatory mediators in the vascular environment. In addition to regulating redox balance and balancing pro- and anti-inflammatory chemicals, ECs in atherosclerotic plaque go through autophagy to maintain NO levels [81].

### 3.2. Autophagy in Vascular Smooth Muscle Cell

Atherosclerotic plaque and extracellular matrix are the primary causes of pathological thickening of the intima of early atherosclerosis, and VSMCs are the most important source of these materials. Moreover, in both humans and mice, VSMCs are found to be a major source of foam cells [82]. A newly published study found that VSMC-specific *HuR* knockdown leads to plaque formation and instability due to deficits in autophagy [83]. Atherosclerosis and stress-induced premature ageing are accelerated by the deletion of *Atg7* in VSMCs. Autophagy is not only crucial for survival but also regulates VSMC phenotypes and functions. Autophagy-inducing factors, such as PDGF-BB, POVPC, and osteopontin, reduced contractile phenotype in VSMCs and motivated proliferation and migration while inhibiting autophagy-promoted contractile phenotype preservation [84,85,86]. In recent evidence, autophagy appears to be involved in the differentiation of VSMCs into foam cells. Through the PI3K-Akt-mTOR pathway, the P2RY12 receptor inhibits autophagy in VSMCs, reducing cholesterol lipolysis and promoting foam cell conversion [87]. Similarly, autophagy flow is significantly reduced in VSMC-derived foam cells, which impedes lipid clearance [82].

### 3.3. Autophagy in Macrophage

The etiology of atherosclerosis is significantly influenced by macrophage (mφ) autophagy, which prevents oxidative stress, inflammation, and the growth of foam cells. Mechanistic research in *Atg5*-macrophage KO mice has demonstrated that disruption of autophagy in macrophages causes a noticeably higher atherosclerosis burden [88]. As well as protecting against apoptosis, macrophage autophagy facilitates the clearance of apoptotic cells through efferocytosis [89]. Efferocytosis occurs when specialized phagocytes (such as macrophages) or unspecialized phagocytes (such as epithelial cells) phagocytose apoptotic cells for repair and homeostasis. The impairment of efferocytosis results in apoptotic cell membrane degrading rapidly, releasing intracellular contents, destabilizing plaques, stimulating angiogenesis, and promoting atherosclerosis through elevated release of thrombotic factors [90,91]. Furthermore, defective efferocytosis signaling inhibits the intracellular cholesterol reverse transportation pathways, promoting foam cell formation [92]. *Atg5*-deficient mice developed plaque necrosis more quickly, suggesting efferocytosis defects in their macrophages [93]. A process known as lipophagy labels lipid droplets (LDs) and clears them via the ubiquitin system [94]. Using lysosomal acid lipases to autophagically degrade lipid droplets, lipophagy prevents cellular lipid accumulation, which may contribute to atherosclerosis prevention [95]. *Atg5* silencing reduced the reverse cholesterol transport in vivo and abrogated the cholesterol efflux to ApoA-I in vitro [96]. In macrophages, apelin-13 inhibits lipid accumulation by enhancing cholesterol efflux, which is achieved through PI3K/BECN1-activated autophagy [97]. Moreover, atypical ATM-mTOR signaling pathways, along with selective autophagy, prevented macrophages from turning into foam cells [98]. These data collectively demonstrate the atheroprotective function of autophagy in ECs, macrophages, and VSMCs (Figure 3).

## 4. CMA in Atherosclerosis

In the absence of CMA, glycolysis and lipid metabolism proteins are not efficiently degraded, resulting in hypercholesterolemia, hypertriglyceridemia, and insulin resistance, all of which are risk factors for atherosclerosis [10]. According to Qiao et al. [11], CMA dysfunction is associated with atherosclerosis, as the expression of LAMP2 gradually decreases over time in high-fat diet-fed mice. Additionally, the macrophage-specific LAMP-2A knockout mouse showed significantly higher lesion areas (52%) and higher serum levels of pro-inflammatory factors IL-18 and IL-1β in atherosclerotic plaques. An analysis of the transcriptional profile of LAMP-2A VSMCs revealed that nodes related to cell proliferation, cell migration, lipid response, and differentiation were upregulated [10]. In addition, a robust pro-inflammatory profile is observed in BMDMs with CMA deficiency (higher iNOS and COX2 levels), indicating that CMA may regulate the polarization of macrophages toward inflammation.

## 5. Mitophagy in Atherosclerosis

The impaired mitophagy in AS contributes to multiple atherosclerotic risk factors, such as endothelial dysfunction, VSMC apoptosis or proliferation, and macrophage polarization, and promotes atherosclerosis development. Endothelial cells produce ATP by glycolysis instead of oxidative phosphorylation, and thus mitochondria serve as signal transduction mediators for paracrine, mobilization, and proliferation signaling [99,100]. The mitochondria of ECs exhibit several types of damage under stress, including reactive oxygen species (ROS) production, the opening of mitochondrial permeability transition pores, and mitochondrial DNA (mtDNA) release [101]. ROS overproduction can trigger EC senescence and promote atherosclerosis by stimulating apoptosis [102]. *Atg5* and *Atg12*, two mitophagy-related genes that are overexpressed in EC and have been shown to increase membrane potential, boost ATP synthesis, have antiapoptotic effects, and increase resistance to oxidative stress [103].

There is a critical role for mitophagy in governing VSMC phenotype, function, apoptosis, and survival. The AMPK/OPA1 pathway activates mitophagy through melatonin, which increases LC3II and MFN2, and diminishes calcium deposition, so VSMCs are protected from vascular calcification [104]. Human VSMCs undergo mitophagy as a safeguard against oxidized LDL-induced apoptosis. As an example, loss of PINK1 and PARK2 increased Caspase activity and oxidized LDL-mediated cell death, thereby supporting their antiapoptotic effect [105]. The excessive mitophagy of VSMCs may also cause abnormal proliferation. PINK1/PARK2-mediated mitophagy promotes apelin-13-induced VSMC proliferation in human aorta and exacerbates the progression of atherosclerosis [106].

A known selective caspase 1 inhibitor, VX765, inhibits migration, pyroptosis, and foam cell formation of macrophages by facilitating mitophagy. Using melatonin, Ma et al. [107] found that Sirtuin 3 (Sirt3)/FOXO3/PARK2 induces mitophagy, resulting in a reduction in mitochondrial ROS production. Macrophages lacking mitochondrial Apolipoprotein A-I binding protein (AIBP) led to mitochondrial metabolism disorders, resulting in more PINK1 protein cleavage and suppression of mitophagy, which ultimately caused macrophages to convert to the M1 type, causing inflammation to worsen [108]. A binding of AIBP to PARK2 enhances the ubiquitination of MFN1 and MFN2, resulting in the formation of autophagosomes and the removal of damaged mitochondria [109]. Nonetheless, mitophagy’s direct contribution to atherosclerosis remains elusive, and further investigation of the co-localization of mitochondrial markers and autophagy markers is needed.

It is thought that autophagy plays an important role in AS at each stage: in the early stages, it may reduce the accumulation of lipids and decrease the formation of foam cells; in the advanced stages, it may remove necrotic cells and retard plaque formation [110]. Autophagy may, however, play a ‘detrimental’ or ‘beneficial’ role in atherosclerosis, since both labels have been associated with the disease. The formation of ceroids, yellow-to-brown insoluble protein complexes associated with oxidized lipids in atherosclerotic lesions, can be attributed to autophagy during severe oxidative stress. Increasing ceroid accumulation in lysosomes disrupts lysosomal hydrolase function, thus favoring ROS production and waxy pigment accumulation and ultimately leading to cell death [111]. In addition, hyperactive autophagy induces apoptosis of SMCs, inducing fibrous membrane formation and reducing normal collagen synthesis, thus deepening plaque instability. It is likely that autophagy’s negative effects are related to its overactivation, which may lead to cell death and exacerbate atherosclerosis. In atherosclerosis, therefore, autophagic activity must be balanced to promote plaque stability.

## 6. Overview of NLRP3 Inflammasome Biology

Microbial pathogens and sterile injury provoke innate immune cells to activate pattern recognition receptors (PRRs) [112]. To date, five families of PRRs have been described, most of which are expressed on leukocyte membranes: NLRs, TLRs, CLRs, RLRs, and ALRs [113]. The ALR and NLR are responsible for recognizing pathogens releasing pathogen-associated molecular patterns (PAMPs) during microbial pathogens infection, as well as danger-associated molecular patterns (DAMPs), as a result of cellular damage. As an NLR, NLRP3 has a PYD domain at the amino terminus, a NACHT domain, and an LRR domain at the carboxy terminus [114]. When activated, the LRR domain oligomerizes NLRP3 via its NACHT domain, whereas the effector PYD is in charge of enhancing downstream pro-inflammatory responses through a PYD–PYD interaction with ASC. Via homotypic CARD–CARD interactions, the assembled ASC recruits pro-caspase-1, forming the NLRP3–ASC–caspase-1 protein complex. The activated NLRP3 inflammasome triggers the activation of caspase 1, leading to the maturation and secretion of inflammatory cytokines, such as IL-18 and IL-1β, and the cleavage of gasdermin D, resulting in the inflammatory cell death process, pyroptosis [115,116,117,118].

### 6.1. NLRP3 Inflammasome Activation

There is intense debate about the mechanisms leading to NLRP3 inflammasome activation, including intracellular K^+^ decreases, mitochondrial ROS (mtROS) production, and lysosome cathepsin release [115,119]. Extracellular ATP stimulates purogenic P2X7 ATP-gated ion channels, triggering K^+^ efflux and gradually recruiting pannexin-1 membrane pores [120]. Additional downstream effects may result from K^+^ flux (e.g., Ca^2+^ flux, mitochondrial disruption, and cell volume regulation). A variety of particles, such as monosodium urate, cholesterol crystals (CCs), asbestos, and silica, are engulfed by phagocytes, causing lysosomal content release that is detected by the NLRP3 inflammasome [121,122]. By releasing mitochondrial DNA (mtDNA) in response to NLRP3 activators, ROS converts it to an oxidized form, which drives the activation of NLRP3 inflammasomes [123,124]. According to a recent study, mtROS may also cause lysosomal damage, which could theoretically trigger the NLRP3 inflammasome activation [125]. Another potential ROS source is lysosomal NADPH oxidase; however, the activation of the NLRP3 is in dispute.

### 6.2. NLRP3 Inflammasome in Atherosclerosis

The expression of NLRP3 in aorta tissue specimens of patients with atherosclerosis has been correlated with the disease’s severity [126]. Notably, the increased expression of inflammasome components, such as caspase-1, NLRP3, ASC, and IL-1β, was detected in atherosclerotic plaques from symptomatic patients [127]. Atherogenesis in animal models has further illustrated the effect of NLRP3 inflammasome. Double knockout mice lacking *Apoe* and *caspase-1* show reduced progression of atherosclerotic lesions [128]. In *Ldlr*^−/−^ mice implanted with *Asc^−^*^/−^, *Nlrp3*^−/−^, or *IL-1β*^−/−^ bone marrow, atherosclerosis and inflammasome-dependent IL-18 levels were significantly reduced relative to mice implanted with wild-type bone marrow, suggesting NLRP3 inflammasome functions are genetically determined [129]. The efflux of cholesterol mediated by inflammasomes is also essential to atherogenesis. The transplantation of bone marrow from mice lacking myeloid *Abca1/g1*, along with having an *Nlrp3* deficiency, into *LdIr*^−/−^ mice fed a western-type diet attenuated atherosclerotic lesions in myeloid *Abca1/g1*-deficient mice [130]. Collectively, epidemiological correlations and experimental evidence increasingly support NLRP3 inflammasome’s role in atherogenesis. The activation of the NLRP3 inflammasome in atherosclerosis is primarily driven by oxidized LDL and cholesterol crystals. In a similar way to other known crystalline NLRP3 activators, CCs activate the NLRP3 inflammasome through lysosomal damage. As CCs are phagocytosed by macrophages, their phagocytosis is inefficient, resulting in lysosomal destabilization and the leakage of cathepsins, activating the NLRP3 inflammasome [129,131]. Acute inflammation can be induced by intraperitoneal injection of cholesterol crystals in WT mice; however, this is not evident in mice lacking NLRP3 inflammasome, cathepsin L, or cathepsin B [129]. Furthermore, recent studies suggest that CCs activate the NLRP3 inflammasome via diverse mechanisms, for example, C5a [132], as well as Nrf2 [133]. The oxLDL, once transported to the lysosomes, triggers the NLRP3 inflammasome by provoking cholesterol crystallization and lysosomal damage. A scavenger receptor on macrophages, CD36, incorporated oxidized LDL into the cells and induced the formation of a TLR4/TLR6 heterodimer, contributing to NF-κB signaling [134]. CCs also act as feed-forward loops by enhancing the expression of CD36, thereby facilitating subsequent oxLDL uptake by macrophages [135]. Taken together, NLRP3 inflammasome is largely responsible for the development of atherosclerosis.

## 7. Autophagy–Inflammasome Interrelation in Atherosclerosis

The progression of atherosclerosis is characterized by lipid accumulation and inflammation, and the balance between proinflammatory and inflammation-resolving processes determines the final outcome. Recent studies suggest that impaired and inhibited autophagy may contribute to SMCs and ECs’ inflammatory responses induced by oxLDL [136]. Inflammasome regulation by selective autophagy is ripe for study. There has been considerable research on the negative regulation of the inflammasome by selective autophagy. Initially, Shi [137] and colleagues described inflammasome regulation by polyubiquitinating Lys63 on the ASC subunit, which is targeted to autophagosomes by SQSTM1/p62, which serves as a primary selective autophagy receptor. Inflammasome components, including NLRP3 and caspase-1, are recognized explicitly by the selective autophagy receptor TRIM20 independent of ubiquitination, serving as a platform to spur on autophagy by recruiting ULK1, BECN1, and ATG8 [138]. Plaque formation in mice was accompanied by an increased co-localization of autophagic markers with macrophages (mϕ). Inflammasome markers were robustly induced in ATG5-null macrophage, particularly when combined with cholesterol crystals [88]. Additionally, it appears that cholesterol crystals are more abundant in ATG5-mKO plaques, indicating a potential vicious cycle between crystal formation and inflammasome activation [88]. Autophagy–lysosome biogenesis is primarily controlled by the transcription factor TFEB, which transcribes a wide range of autophagy–lysosomal genes. The overexpression of TFEB in macrophages reversed the autophagy–lysosomal impairments caused by atherogenic lipids. It also showed several beneficial functional effects, including regulating cholesterol efflux and restraint inflammasome activity, as well as removing aggregates of polyubiquitinated proteins [139]. Defective autophagy contributes to the hyperactivation of NLRP3 inflammasomes mediated by cholesterol crystals [8]. Additionally, autophagy deficiency has inflammatory effects specific to the inflammasome, since other pro-inflammatory cytokines, such as TNF-ɑ and IL-10, are not affected [88]. A recent study has indicated that nicotinamide adenine dinucleotide-dependent sirtuin 3(SIRT3), a deacetylase sensitive to metabolic stimulation, facilitates autophagy deficiency and NLRP3-mediated atherosclerosis A recent study has indicated that nicotinamide adenine dinucleotide-dependent SIRT3, a deacetylase sensitive to metabolic stimulation, hinders autophagy deficiency and NLRP3-mediated atherosclerosis [140]. Liu et al. [141] discovered that SIRT3 forms a molecular complex with ATG5. In peripheral monocytes from obese adults, SIRT3 levels were reduced, which resulted in NLRP3 inflammasome activation and autophagy obstruction.

**Figure 4:**
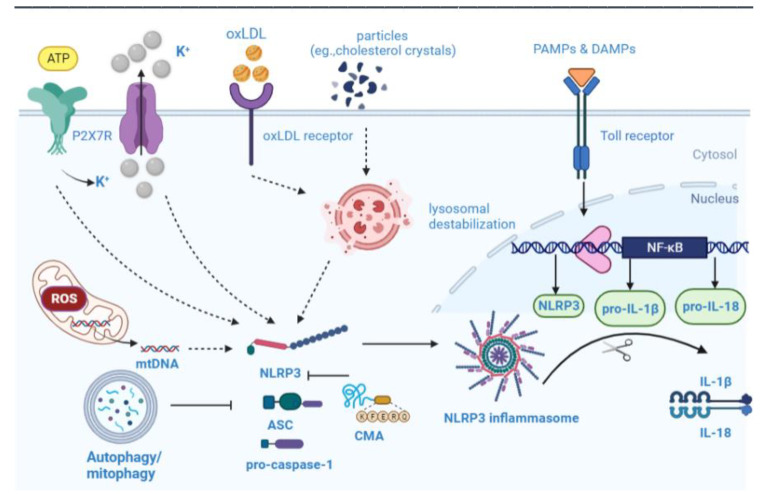
The mechanisms of the effects of autophagy on NLRP3 inflammasome. NLRP3-activating signals, such as cholesterol crystals, cause damage to lysosomes and mitochondria, resulting in cathepsin release, ROS release, and K+ excretion. It leads to the assembly and activation of inflammasomes, followed by the formation of active caspase-1. Inflammation is aided by active caspase-1, which converts pro-forms of IL-1β and IL-18 into their mature forms. Mitophagy and autophagy are both able to decrease mtROS production by clearing the damaged mitochondria, thus inhibiting NLRP3 inflammasomes. In addition, the NLRP3 protein was discovered to be a substrate for chaperone-mediated autophagy and was degraded into the lysosome by this pathway. The arrows represent the promotion, and the blunt arrows represent the inhibition.

By scavenging mtROS, mitophagy serves as a regulator of NLRP3 inflammasome activation [142,143]. The activated inflammasome is regulated by the disruption of mitochondrial integrity and dysregulation of autophagic clearance, which releases ROS and mtDNA from mitochondria [143,144]. The caspase 1-inhibitor VX765 facilitates mitophagy and M2 polarization of macrophages, alleviating either vascular inflammation or atherosclerosis in *Apoe^−/−^* or *Ldlr^−/−^* mice [145]. The abolition of NLRP3 in *Apoe^−/−^* mice, however, abrogated the effects of VX765. Several proteins involved in lipid metabolism are bona fide CMA substrates, including lipogenesis enzymes and lipid droplet coat proteins [146,147]. Additional regulatory roles for CMA have been identified beyond metabolic pathway control. CD4^+^ T cell activation depends on CMA, which degrades two negative regulators of TCR signalling [148]. CMA-incompetent T cells display persistently high levels of these two factors, which results in reduced proliferation and cytokine release. This observation reveals a link between immune response and CMA activity. Chronic inflammatory diseases, such as atherosclerosis, impair CMA activity when exposed to pro-atherosclerotic challenges. In VSMCs, CMA loss promotes de-differentiation and increased lipid sensitivity, whereas macrophages with defective CMA become more pro-inflammatory [10,148]. Serum cholesterol and triglyceride levels did not differ statistically significantly between LAMP-2A- mKO/*Apoe^−/−^* and WT mice, but serum IL-18 and IL-1β levels were elevated in LAMP-2A- mKO mice [11]. In an analysis of CMA-lysosome degradation of NLRP3, it was identified as a substrate of the CMA. LAMP-2A deficiency prevented NLRP3 protein breakdown through CMA but did not alter NLRP3 mRNA levels [11]. Moreover, it remains to be confirmed whether CMA promotes the degradation of the other components of NLRP3 inflammasome activation (Figure 4). The NLRP3 inflammasome is expected to be a therapeutic target for atherosclerosis. Through several mechanisms, autophagy negatively regulates the activation of NLRP3 inflammatory vesicles, maintaining cellular homeostasis. Hence, enhancing autophagy to lessen inflammatory responses can alleviate AS.

## 8. Pharmacological Opportunities in Autophagy–Inflammasome Interplay

In light of the evidence indicating that autophagy defects increase NLRP3 activity, several autophagy regulators have been found to inhibit NLRP3 inflammasomes, attenuating AS. For example, to alleviate atherosclerosis, fucoidan (a polysaccharide mainly composed of fucose and sulfate) inhibits NLRP3 activation through SQSTM1/p62-dependent selective autophagy [149]. FDA-approved miltefosine promotes cholesterol release and disrupts lipid rafts for controlling visceral and cutaneous leishmaniasis. The cells treated with miltefosine showed an increase in autophagy-inducing kinases ULK1 and AMPK1, which resulted in an increase in basal autophagy and mitophagy, as well as a blunting effect on ROS generation and NLRP3 inflammasome assembly by lipopolysaccharide (LPS) [150]. Additionally, autophagy contributed to dietary PUFA-mediated atheroprotection by restraining the generation of NLRP3-related pro-inflammatory cytokines [151]. A potent inhibitor of farnesyl transferase, arglabin, exerts anti-inflammatory and anti-atherosclerotic effects on AS [152]. Melatonin inhibits the NLRP3 inflammasome via mitophagy, which reduces the size and vulnerability of AS plaques [107]. A newly synthesized compound, 13-methylberberine, suppresses the activation of NLRP3 inflammasomes and enhances autophagy to alleviate endothelial dysfunction [153]. Rivaroxaban inhibits the factor Xa-protease-activated receptor 2-mediated suppression of macrophage autophagy and abrogates inflammasome activity during atherogenesis [154]. As the main active ingredient in the traditional Chinese herb *Clematis chinensis*, Clematichinenoside AR (AR) may be a promising therapeutic agent in the treatment of AS. It inhibits NLRP3 activity primarily by regulating autophagy, promotes cholesterol efflux, and inhibits foam cell formation [155]. The statin drug atorvastatin, commonly used for hypercholesterolemia and atherosclerosis, has intriguing anti-inflammatory properties in addition to its lipid-lowering abilities. By inducing autophagy, atorvastatin reduces the vulnerability of atherosclerosis plaques in mice, inhibits the inflammatory response, and activates the NLRP3 inflammasome, as well as inhibiting foam cell formation and inflammatory cytokine secretion in macrophages stimulated with ox-LDL [156]. Specifically, studies shed light on how autophagy regulates atherosclerosis and provides potential therapy options for the prevention and treatment of atherosclerosis. Herein, several typical pharmacological inhibitors targeting the development of AS are discussed below (Table 1).

## 9. Conclusions

The review summarized the current studies associated with autophagy and NLRP3 inflammasome in atherosclerosis. The progression of atherosclerosis is influenced by autophagy on endothelial cells, VSMCs, and macrophages; it affects, for example, VSCM proliferation, migration, phenotype switching, endothelial function, lipid metabolism, and inflammation, which are linked with the formation and progression of atherosclerotic plaques. A decrease in autophagic flux or impaired autophagy may result in mitochondrial disruption, ROS production, and lysosomal destruction in vivo. The NLRP3 inflammasome is further activated by protein aggregates and intracellular release factors. Experimental evidence and epidemiological correlations point to the role of the NLRP3 inflammasome in atherogenesis. By using ATG knockout mouse models, many studies have identified autophagy as a limiting factor on NLRP3 inflammasome. The autophagy process can isolate and degrade inflammasome components, such as pro-IL-1β, caspase-1, ASC, and NLRP3. Multiple studies have shown that NLRP3 inflammasome activation regulates the induction of autophagy, while autophagy also controls inflammasome activation. Autophagy modulators that interfere with NLRP3 inflammasomes are now being discussed pharmacologically as a potential treatment for AS in several studies. As a consensus model has not yet been developed, more experiments, especially clinical trials, are desperately needed. Further investigation is required to determine whether targeted autophagy/NLRP3 inflammasomes can be effective in treating AS, and other research is required to identify the best drug candidates and delivery systems.

## Figures and Tables

**Table 1:** Antiatherosclerotic compounds and mechanisms.

Compounds	Mechanisms of Autophagy Induction	Antiatherosclerotic Effects	References
Fucoidan	Autophagy (direct)	↓NLRP3 inflammasome ↓lipid accumulation	[149]
Miltefosine	AMPK/ULK1	↓mtROS ↑mitochondrial membrane potential ↓NLRP3 inflammasome ↑cholesterol release	[150]
PUFAs	Autophagy (direct)	↓NLRP3 inflammasome ↓IL-1β secretion caspase-1 cleavage	[151]
Arglabin	Autophagy (direct)	↓IL-1β and IL-18 ↓plasma lipids ↑anti-Inflammatory M2 Phenotype	[152]
Melatonin	Sirt3/FOXO3a/Parkin	↓mtROS ↓NLRP3 inflammasome ↓plaque size and vulnerability	[107]
VX765	*Parkin*	↓NLRP3 inflammasome assembly ↓foam cell formation ↑efferocytosis	[145]
13-Methylberberine	Autophagy (direct)	↓ROS ↓NLRP3 inflammasome	[153]
Rivaroxaban	Protease-activated receptor 2	↓NLRP3 inflammasome atherosclerotic plaques↓	[154]
Clematichinenoside AR	Autophagy (direct)	↓NLRP3 inflammasome ↑ABCA1/ABCG1 ↑cholesterol efflux ↓ foam cell formation	[155]
Atorvastatin	mTOR	↓NLRP3 inflammasome ↓lipid deposition ↓foam cell formation	[156]

Note: ↑ Represents up-regulation; ↓ Represents down-regulation.

## Data Availability

Not applicable.

## Abbreviations

Atherosclerosis (AS); interleukin-1β (IL-1β); chaperone-mediated autophagy (CMA); endothelial cell (EC); oxidized-low-density lipoprotein (ox-LDL); vascular smooth-muscle cells (VSMCs); Toll-like receptor (TLR); Canakinumab Anti-Inflammatory Thrombosis Outcome Study (CANTOS); mTOR complex 1 (mTORC1); PI3K-related protein kinase (PIKK); tuberous sclerosis complex (TSC); GTPase-activating protein (GAP); Tre2-Bub2-Cdc16 (TBC); pre-autophagosomal structure (PAS); activating molecule in BECN1-regulated autophagy protein 1 (AMBRA1); PtdIns III K produces phosphatidylinositol-3-phosphate (PtdIns3P); WD repeat domain phosphoinositide-interacting (WIPI); soluble N-ethylmaleimide-sensitive factor attachment protein receptor (SNARE); lysosome-associated membrane protein type 2A (LAMP-2A); endosomal microautophagy (eMI); endosomal sorting complex required for transport (ESCRT); inner mitochondrial membrane (IMM); outer mitochondrial membrane (OMM); optineurin (OPTN); light chain 3B (LC3B); BCL2 interacting protein 3 (BNIP3); NIP3-like protein X (NIX); Bcl2-like protein 13 (Bcl2-L-13); FUN14 domain-containing protein 1 (FUNDC1); GABA-receptor-associated protein (GABARAP); hypoxia-inducible factor-1 (HIF-1); forkhead homeobox type O (FOXO); nitric oxide (NO); lipid droplets (LDs); reactive oxygen species (ROS); mitochondrial DNA (mtDNA); Sirtuin 3 (Sirt3); Apolipoprotein A-I binding protein (AIBP); pattern recognition receptors (PRRs); pathogen-associated molecular patterns (PAMPs); danger-associated molecular patterns (DAMPs); caspase-1 (CASP1); mitochondrial ROS (mtROS); cholesterol crystals (CCs).
